# Folliculosebaceous Cystic Hamartoma With Unusual Mucinosis: A Report of a Rare Case

**DOI:** 10.7759/cureus.83792

**Published:** 2025-05-09

**Authors:** Riya Patel, Jaime Tschen

**Affiliations:** 1 Department of Dermatology, UTHealth Houston McGovern Medical School, Houston, USA; 2 Department of Dermatology, St. Joseph Dermatopathology, Houston, USA

**Keywords:** benign cutaneous lesion, clinical dermatology, dermal mucinosis, dermatopathology, follicular mucinosis, folliculosebaceous cystic hamartoma, skin disease/dermatology

## Abstract

We report a case of a clinically indistinct pink papule on the midline upper back of a 77-year-old woman. Histopathological examination of the lesion revealed a unique variant of folliculosebaceous cystic hamartoma (FSCH) characterized by mucin deposits in sebaceous lobules. FSCH is a rare lesion involving a cystically dilated follicle surrounded by sebaceous glands embedded within a stromal matrix composed of mesenchymal features. Follicular mucinosis is an uncommon inflammatory disorder typically presenting as benign, idiopathic lesions in children or mycosis fungoides-associated lesions in adults. A literature review revealed no previously reported cases of FSCH involving mucinosis of our knowledge to date, making this a novel and clinically relevant histopathologic variant. This case expands the histological presentation of a rare lesion and highlights the importance of careful examination in differentiating between similarly presenting dermatological conditions. Additionally, this novel finding suggests the possibility of an unknown pathologic mechanism of mucin deposition in FSCH, warranting further research.

## Introduction

Folliculosebaceous cystic hamartoma (FSCH) is a rare, benign cutaneous hamartoma first described by Kimura et al. in 1991 [1]. The typical presentation is a slow-growing, individual, flesh-colored papule or nodule and is composed of follicular, sebaceous, and mesenchymal components. Most commonly, the lesion presents on the face or scalp as a dome-shaped or pedunculated nodule, but rare presentations involving the genitals, earlobes, and upper back have been reported [2]. Although rare cases of giant FSCH have been reported, the lesions are typically less than two centimeters [3-5]. The vague clinical presentation of FSCH yields many differential diagnoses, including epidermal inclusion cyst, sebaceous hyperplasia, mycosis fungoides (MF), basal cell carcinoma, and sebaceous trichofolliculoma. Due to indistinct clinical features, the evaluation of FSCH must rely on careful histological examination to appropriately guide diagnosis and treatment regimens. The classic histologic presentation of FSCH is well established, with the first description appearing in Kimura et al. However, mucin deposition, as exhibited in our case, is not an associated feature. Follicular mucinosis, defined as the accumulation of dermal-type mucin in the pilosebaceous unit, is a rare epithelial reaction pattern. There are two forms of follicular mucinosis typically recognized: a primary (idiopathic) form typically affecting children and young adults with a benign course, and a secondary form associated with cutaneous T-cell lymphoma (CTCL), particularly MF, and other inflammatory conditions [6].

This case was presented as a virtual poster at the Spring 2025 Texas Dermatological Society meeting.

## Case presentation

A 77-year-old woman presented to her dermatologist with a clinically indistinct pink papule on her midline upper back. There were no other lesions suggestive of follicular mucinosis present, and the patient did not have a history of MF. A 0.8 × 0.7 cm shave biopsy was collected by her dermatologist from the lesion, and possible differential diagnoses included an epidermal inclusion cyst. Atypical cellular features were noted, prompting further dermatopathological examination. Detailed histopathologic examination revealed findings consistent with FSCH with prominent mucus deposition - an uncommon presentation of this rare lesion. The biopsy focally involved the deep margin, suggesting the entire lesion may not have been excised. No further clinical information or follow-up was available.

**Figure 1:**
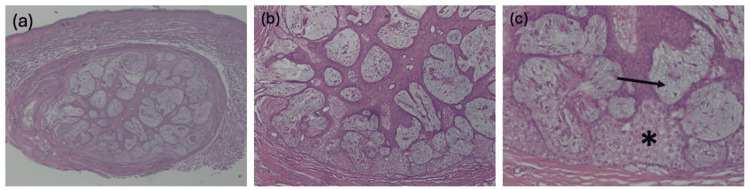
Hematoxylin and eosin (H&E) staining of the lesion obtained via shave biopsy at increasing magnifications. (a) The lesion is a well-circumscribed proliferation of sebaceous glands embedded in a stromal matrix, characteristic of a folliculosebaceous cystic hamartoma (H&E, 20x). (b) Higher power view reveals no cytologic atypia or cellular abnormalities (H&E, 40x). (c) The lesion demonstrates abundant mucus deposition (black arrow) within sebaceous lobules. Normal sebaceous cells are marked with an asterisk (H&E, 100x).

**Figure 2:**
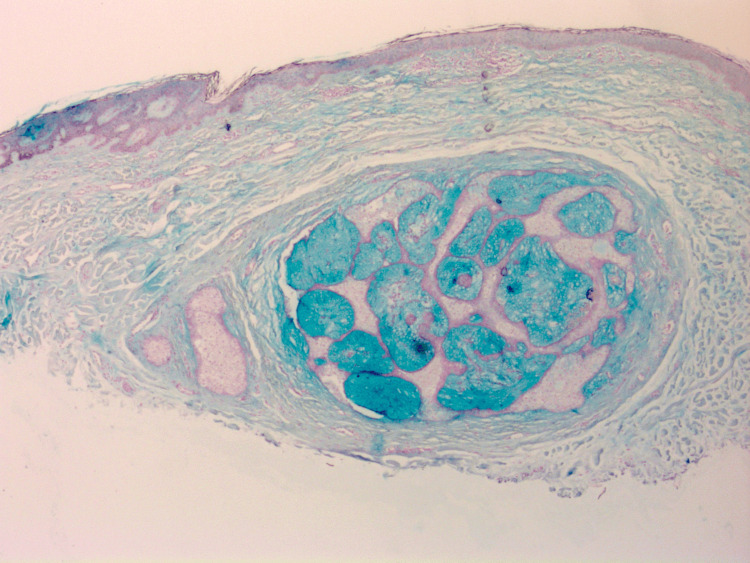
Alcian blue staining confirms the abnormal accumulation of mucin in sebaceous glands, atypical for folliculosebaceous cystic hamartomas (Alcian blue, 20x). Global brightness and contrast adjustments were made to improve image clarity.

**Figure 3:**
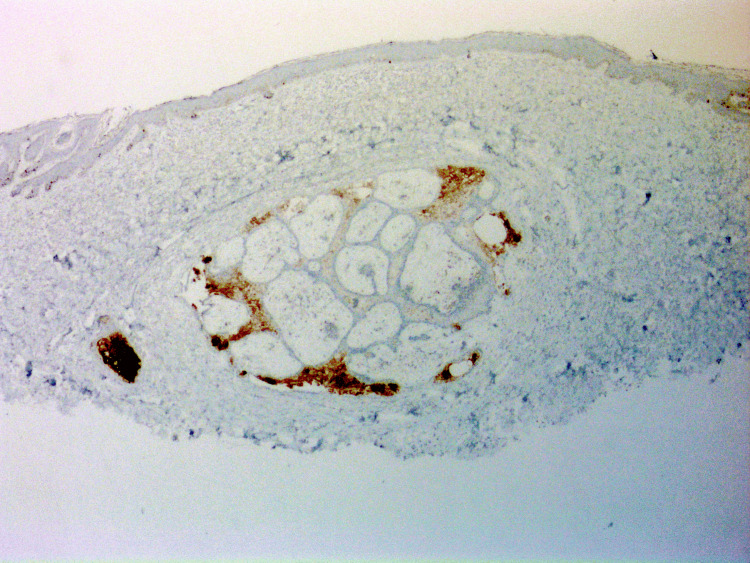
PRAME positivity confirms the lesion is of sebaceous origin, differentiating it from other mucinous neoplasms (PRAME, 20x). Global brightness and contrast adjustments were made to improve image clarity. PRAME: Preferentially Expressed Antigen in Melanoma

Upon histologic examination, the lesion demonstrated features characteristic of FSCH, including a proliferation of sebaceous lobules embedded in a mesenchymal stroma composed of fibrous tissue, adipocytes, and small vessels (Figure 1a). Additionally, the lesion did not demonstrate any cytologic atypia, confirming the diagnosis of a benign lesion (Figure 1b). Inconsistent with the typical histologic presentation of FSCH, the lesion demonstrated prominent mucin deposition within sebaceous lobules (Figure 1c). The mucin deposition was further confirmed by Alcian blue staining (Figure 2). The remaining glands are confirmed to be of sebaceous origin by positive Preferentially Expressed Antigen in Melanoma (PRAME) staining (Figure 3).

## Discussion

This case emphasizes the importance of precise histologic examination in diagnosing clinically indistinct dermatoses. FSCH often presents as a nonspecific papule or nodule, making clinical diagnosis challenging without microscopic evaluation. In this case, despite the presence of classic histologic features representative of FSCH, an additional, atypical finding of mucin deposition within sebaceous lobules was observed.

To contextualize this patient’s unique presentation, we reviewed reported cases of FSCH in the literature and compiled a comparison of patient and lesion characteristics. A focused literature review was conducted using PubMed Central to identify open-access case reports with a confirmed primary diagnosis of FSCH for further comparison. This search yielded 12 PubMed Central-indexed case reports with a primary diagnosis of FSCH and freely available full text. These cases were reviewed for available data on patient age, sex, lesion location, lesion size, and key features (Table 1). Our case is notable for the lesion's location on the upper back and histologic presence of mucin in sebaceous glands - features not observed in any of the 12 analyzed cases.

**Table 1:** Comparison of reported cases of FSCH with available clinical characteristics. Cases were identified through a PubMed Central search of open-access case reports with a primary diagnosis of FSCH. The search was performed using the terms “folliculosebaceous cystic hamartoma” and “FSCH,” with no date restrictions. Data extracted includes patient age, patient sex, lesion location, lesion size, and any unique or distinguishing findings. FSCH: Folliculosebaceous cystic hamartoma

Author (Year)	Patient Age/Sex	Lesion Location	Lesion Size	Unique Features
Emsen and Livaoglu (2007) [3]	2/F	Posterior leg	7.6 cm	Giant FSCH
Haw and Lee (2009) [4]	48/F	Lower cheek	10×8×5 cm	Giant FSCH
Tasar et al. (2023) [5]	84/M	Side of face	26.0×11.0 cm	Giant FSCH
Noh et al. (2011) [7]	38/M	Earlobe	0.8×1.2 cm	Misdiagnosed as neurofibroma
Watanabe-Okada et al. (2014) [8]	29/M	Nose	1.5 cm	-
Nguyen et al. (2015) [9]	24/M	Nose	0.6 cm	Spindle cell lipoma-like stromal features
Rajamohanan et al. (2017) [10]	45/M	Malar area, adjacent to nose	0.7×0.7 cm	-
Bobde et al. (2019) [11]	1/F	Posterior thigh	10×8×5 cm	Congenital variant
Carnevale et al. (2021) [12]	Newborn/M	Paravertebral lumbosacral area	8×5×3.5 cm	Congenital variant
Subasi et al. (2021) [13]	17/F	Preauricular	2×1 cm	Preauricular region, located subcutaneously
Daruish and Ibrahim (2022) [14]	35/F	Labia majora	0.5-2 cm	Multiple lesions, genital FSCH
Palomino Aguilar et al. (2024) [15]	28/F	Scalp	0.7×0.6×0.4 cm	-

Upon histopathologic examination, FSCH is classified as a cystically dilated infundibular structure with radiating sebaceous lobules. This proliferation is embedded in a stromal matrix of mesenchymal elements including collagen, adipocytes, mucin, and blood vessels [16]. Rare histologic variants of FSCH have been reported in the literature, including lesions with melanocytic, neural, vascular, and spindle-cell lipoma-like components [7,9,17]. Notably, a case of FSCH with perifollicular mucinosis was described by Aloi et al. in 1996, in which large amounts of mucin were identified in the stroma surrounding the lesion [18]. In contrast, our case demonstrates significant mucin accumulation specifically within sebaceous lobules in association with FSCH. Thus, to our knowledge, this represents a distinct and previously unreported histopathologic finding.

The accumulation of mucin within sebaceous lobules in this lesion necessitates comparison to other cutaneous conditions involving mucin deposition. Follicular mucinosis, in particular, is a rare condition characterized by the accumulation of dermal-type mucin within the external root sheath and sebaceous glands. This definition stems from Pinkus' original 1957 description of six cases, in which certain cases demonstrated mucin deposition in both the follicular epithelium and sebaceous structures [19]. Subsequent case reports have confirmed that this dermatosis typically presents in two forms. The primary form is idiopathic, benign, and most often occurs in young individuals without an association with MF. The secondary form presents in adults and is often linked to CTCL, particularly MF. The pathogenesis of follicular mucinosis remains unclear, with proposed mechanisms involving cellular alterations leading to mucin production or a cell-mediated immune response [20].

The presence of mucinosis in our reported case of FSCH does not align with either of these typical forms of follicular mucinosis. Thus, mucinosis associated with FSCH represents an atypical presentation, broadening the histopathologic presentation of FSCH, increasing diagnostic considerations, and suggesting the possibility of an abnormal pathogenesis. This unique presentation warrants further investigation and raises several hypotheses regarding the nature of this novel finding. These include the possibility of a distinct histologic variant of FSCH with associated mucinosis in sebaceous glands, or that the mucin deposition within sebaceous glands may represent a reactive phenomenon secondary to the underlying hamartomatous proliferation. Further exploration of the underlying pathogenesis of mucin deposition in cutaneous lesions, including cytokine-mediated mucin production, cell-mediated immune responses, or sebocyte-epithelial interactions, may help explain this unique presentation.

FSCH is a benign lesion with histologic examination demonstrating a lack of cellular atypia. Consistent with this finding, simple surgical excision is sufficient in removing the lesion while preventing recurrence. To date, malignant transformation of FSCH has not been reported, and there is no need for routine follow-up. In contrast, the standard of care for lesions demonstrating follicular mucinosis is significantly different. Due to the association of follicular mucinosis with malignant or aggressive conditions, routine clinical follow-up, typically for a minimum of five years, is required to monitor disease progression. In cases of secondary follicular mucinosis, treatment of the underlying cause, often MF, is necessary to resolve symptoms [20]. To our knowledge, the presence of mucin within sebaceous lobules in this case of FSCH does not alter the clinical prognosis or recommended management of the lesion. Rather, this report aims to highlight a unique histopathologic finding that may aid dermatopathologists and clinicians in distinguishing FSCH from other cutaneous conditions involving mucin deposition. Additional case reports are essential to further understand the clinical relevance and potential implications of this histologic variant.

## Conclusions

Accurate prediction of prognosis, risk of recurrence, and treatment options for clinically indistinct cutaneous lesions such as FSCH depends on precise diagnosis through careful histologic examination. We describe this case to highlight a rare histopathologic variant of FSCH. This case is notable for demonstrating solitary mucinosis within sebaceous lobules in association with FSCH, a phenomenon that, to our knowledge, has not been previously reported in the literature. Recognizing the presence of coexisting mucinosis in FSCH is important, as this rare combination may histologically mimic malignant conditions such as cutaneous lymphoma. Given the significant differences in prognosis, progression, and treatment between these entities, accurate differentiation is essential. This case highlights the characteristic histologic features and typical presentations of both FSCH and follicular mucinosis to aid clinicians and pathologists in making this distinction. Although other cases of FSCH with mucinosis may exist, the lack of documentation in the literature makes recognition and management challenging. We present this unique finding to increase awareness among clinicians and dermatopathologists that mucinosis within sebaceous glands may present in cases of FSCH. Additionally, this previously unreported presentation sheds light on the necessity of further research on the presentations, reactive responses, and causes of mucin deposition and FSCH.
